# Association Between Lumbar Spinal Stenosis and Accelerated Biological Aging Estimated by PhenoAge

**DOI:** 10.3390/jcm14217852

**Published:** 2025-11-05

**Authors:** Norihiro Isogai, Haruki Funao, Ryo Mizukoshi, Keirato Ito, Shigeto Ebata, Mitsuru Yagi

**Affiliations:** 1 Department of Orthopaedic Surgery, School of Medicine, International University of Health and Welfare, Chiba 286-8520, Japan; n.isogai0813@gmail.com (N.I.); hfunao@yahoo.co.jp (H.F.); masterplan.1651@gmail.com (R.M.); keitaro0518@gmail.com (K.I.); ebatas310@gmail.com (S.E.); 2 Department of Orthopaedic Surgery, International University of Health and Welfare Hospital, Tochigi 329-2763, Japan; 3 Department of Orthopaedic Surgery, International University of Health and Welfare Narita Hospital, Chiba 286-8520, Japan

**Keywords:** PhenoAge, Phenotypic age acceleration, lumbar spinal stenosis

## Abstract

**Background/Objectives**: PhenoAge utilizes biochemical biomarkers to differentiate mortality risk in persons of the same chronological age. However, the details of the relationship between PhenoAge and lumbar spinal stenosis (LSS) remain unclear. We investigated the association between lumbar spinal stenosis (LSS) and biological age quantified by PhenoAge and PhenoAge acceleration (PhenoAgeAccel), comparing surgically treated patients with age- and BMI-matched controls. **Methods**: This study included 208 LSS patients who underwent surgery. The patients were categorized into four subgroups based on gender and age (≥70 years) at the time of surgery. Demographic data, blood biomarkers, body composition measurements, and Phenotypic age acceleration (PhenoAgeAccel), which was assessed by calculating the residuals from regressing PhenoAge on chronological age were compared among the groups. We also compared control groups matched for age and body mass index (BMI) for each of the four groups using medical examination data. **Results**: The mean age was 70.2 ± 9.3 years and the mean PhenoAgeAccel was −5.7 ± 6.5 years in the LSS group. PhenoAgeAccel was significantly lower in the control group (−8.5 ± 3.7 years) than in the LSS group, especially in young male (LSS: −2.9 ± 6.7, Control: −7.0 ± 2.8 years), old male (−4.8 ± 4.4, −6.7 ± 4.0 years), and old female (−6.9 ± 5.9, −10.8 ± 3.2 years) subgroups. In the correlation coefficient between PhenoAgeAccel and BMI, there were weak positive correlations (CC: 0.08–0.31) across all subgroups in the control group, whereas there was a weak negative correlation (CC: −0.29) in the old female subgroup in the LSS group. **Conclusions**: The impact of LSS on PhenoAgeAccel varied by age and gender, and the adverse effect of LSS could be particularly pronounced in elderly women with low BMIs.

## 1. Introduction

Aging is defined as a progressive loss of physiological integrity leading to functional impairment and an increased likelihood of death, and it involves a diverse set of biological changes accumulating over time that lead to increased risk of morbidity and mortality [1,2,3]. Several epigenetic clocks are now widely used to quantify biological aging in order to investigate determinants that modify the rate of aging and to predict age-related outcomes [3,4,5,6,7,8].

PhenoAge utilizes biochemical biomarkers to differentiate mortality risk among individuals of the same chronological age [8]. Previous studies have demonstrated associations between PhenoAge and various diseases, including cancers, diabetes, cardiovascular diseases, depression, and musculoskeletal disorders [9,10,11,12,13,14,15]. PhenoAge, derived from nine biochemical markers and chronological age, has been validated as a robust predictor of frailty and mortality. In patients with lumbar spinal stenosis (LSS), chronic inflammation, sarcopenia, and reduced mobility may promote systemic biological aging [16]. We therefore hypothesized that LSS is associated with higher PhenoAge acceleration (PhenoAgeAccel), particularly in elderly women with reduced skeletal muscle mass.

LSS is one of the most common spinal degenerative diseases and is associated with functional decline and increased mortality risk [16]. Regarding the association between LSS and mortality, appropriate surgical treatment has been reported to significantly reduce mortality [17]. In addition, several studies have demonstrated the high prevalence of both LSS and sarcopenia, which is defined as a low skeletal muscle mass index (SMI) and is associated with reduced life expectancy [18,19]. However, it remains unclear whether this relationship differs by sex, age, or body composition such as skeletal muscle mass. Although elevated PhenoAge has been reported in patients with LSS [15], the details of this association remain insufficiently investigated.

Body weight is tightly linked to LSS occurrence, and its influence should be adequately incorporated into the analysis of treatment outcomes and epidemiology of LSS [20,21]. Underweight, as well as obesity, is also associated with higher mortality [22]. It has recently been reported that both underweight and obesity are similarly associated with higher PhenoAge [23]. Therefore, to investigate the relationship between LSS and PhenoAge, it is critically important to take body weight into account. In this study, we aimed to compare PhenoAge and PhenoAge acceleration between patients with LSS and body mass index (BMI)- and age-matched controls, and to explore their relationship with body composition variables, including BMI and SMI.

## 2. Materials and Methods

### 2.1. Study Design and Participants

This was a retrospective study conducted between 2020 and 2024 at a single hospital in Japan. The study was approved by the institutional ethics committee review board. We investigated LSS patients who underwent decompression or spinal fusion surgery during the period. We excluded patients who had previously undergone spine surgery.

The patients were categorized into four subgroups based on gender and age at the time of surgery. Patients younger than 70 years were classified as the “young” group, while those aged 70 years or older were classified as the “old” group. Subsequently, the cohort was divided into four subgroups: young males, young females, old males, and old females.

Demographic data, blood biomarkers, and body composition measurements were obtained preoperatively. Blood biomarkers including albumin, creatinine, glucose, C-reactive protein (CRP), lymphocyte percentage among white blood cells, mean corpuscular volume (MCV), red cell distribution width–coefficient of variation (RDW-CV), alkaline phosphatase (ALP), and total white blood cell count were measured. PhenoAge was calculated based on the method proposed by Levine et al. using these blood biomarkers [8]. We also evaluated Phenotypic age acceleration (PhenoAgeAccel) which represents the difference between an individual’s PhenoAge and chronological age and is used to quantify the rate of aging [24]. Body composition measurements included BMI and SMI.

### 2.2. Comparison Between LSS and Control

To exclude the impact of BMI on the relationship between LSS and PhenoAgeAccel, control groups matched for age and BMI were established for each of the four subgroups using medical examination data. The control subjects were healthy individuals who underwent routine health check-ups at our institution between 2020 and 2023. Controls were selected from the same institutional health check database and matched for age and BMI. Because information on comorbidities, medications, and activity level was unavailable, these variables were not included in the matching process. Nearest neighbor matching within a caliper was applied based on age (±1 years) and BMI (±0.5 kg/m^2^). For each LSS subject, one control was randomly selected from among candidates meeting these criteria. The final control group was further adjusted to minimize the difference in mean age and BMI compared to the LSS group, ensuring that the average difference in age and BMI between the groups was within ±1.0 years on age and ±1.0 kg/m^2^ on BMI in each subgroup.

Each measured variable was compared across the four subgroups from both LSS and control groups. In addition, correlation coefficients between PhenoAgeAccel and each measured variable were compared across the groups using Fisher’s z-transformation.

### 2.3. Statistical Analysis

We examined whether the associations between PhenoAgeAccel and conventional clinical variables (Age, PhenoAge, SMI, Height, Weight, and BMI) differed across the four demographic subgroups (young male, old male, young female, and old female). For each variable, we computed Pearson’s correlation coefficients with PhenoAgeAccel within each subgroup. Analysis of variance (ANOVA) was applied to evaluate differences among the four subgroups. The association between PhenoAgeAccel and BMI within each subgroup was further analyzed using restricted cubic spline regression. The normality of variables was assessed using the Shapiro–Wilk test prior to applying parametric tests. When multiple subgroup comparisons were conducted, *p*-values were adjusted using the Bonferroni correction. *p*-values < 0.05 were considered statistically significant in these analyses. All statistical analyses were performed using Excel (Microsoft Corp., Redmond, WA, USA) and Python (version 3.12, Python Software Foundation, Wilmington, DE, USA).

## 3. Results

### 3.1. Descriptive Statistics

A total of 208 patients with LSS, including 105 males and 103 females, and 196 participants in the control group, including 103 males and 93 females, were enrolled in this study. The participant demographic data by study groups are summarized in Table 1. The mean age was 70.2 ± 9.3 years ranging from 42 to 90 years in the LSS group. The mean PhenoAge was 65.0 ± 10.7 years ranging from 35.2 to 87.6 years, and the mean PhenoAgeAccel was −5.7 ± 6.5 years ranging from −28.0 to +24.2 years in the LSS group. The mean PhenoAge in the LSS group was higher than that in the control group (62.0 ± 9.2 years), and the mean PhenoAgeAccel in the LSS group was higher than that in the control group (−8.5 ± 3.7 years).

### 3.2. Demographic Data

**Figure 1:**
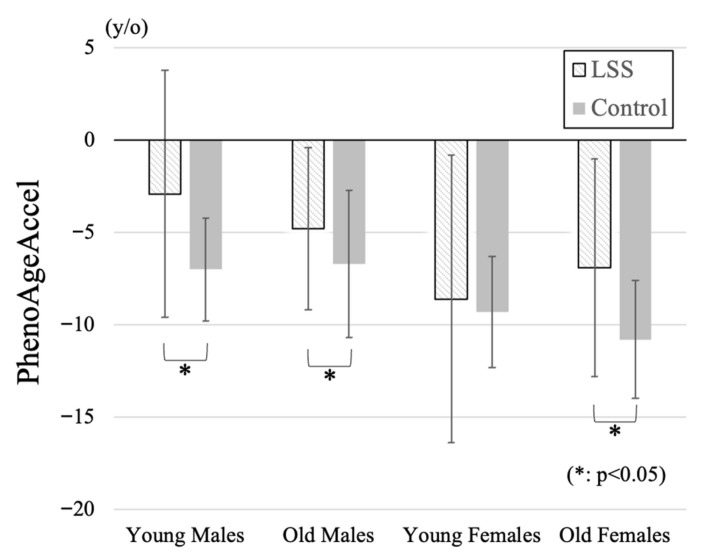
Comparison of PhenoAgeAccel among four subgroups. Positive values indicate higher PhenoAgeAccel (reflecting relatively accelerated biological aging), and negative values indicate lower PhenoAgeAccel (reflecting relatively decelerated biological aging). PhenoAgeAccel was significantly higher in the LSS group compared with controls across the young male, old male, and old female subgroups, indicating accelerated biological aging particularly in elderly women.

Comparison of participant demographic data by four subgroups in the LSS group and the control group was summarized in Table 2. PhenoAgeAccel in young and old females was significantly lower than that in both young or old males. Although there were no significant differences among patient groups in BMI, SMI in old females was significantly lower than those in the other groups. PhenoAgeAccel was significantly higher in the LSS group in young male (LSS: −2.9 ± 6.7, Control: −7.0 ± 2.8 years), old male (−4.8 ± 4.4, −6.7 ± 4.0 years), and old female (−6.9 ± 5.9, −10.8 ± 3.2 years) subgroups (Figure 1). Negative PhenoAgeAccel values represent biologically younger profiles compared with chronological age, whereas less negative or more positive values indicate relatively accelerated biological aging. All subgroup comparisons were statistically validated using Bonferroni-adjusted tests, and only significant differences were interpreted.

### 3.3. Blood Biomarkers

The participant blood biomarker data to calculate PhenoAge by study groups are summarized in Table 3. Serum glucose, C-reactive protein (CRP), lymphocyte fraction, red blood cell distribution width (RDW-CV), alkaline phosphatase (ALP), and white blood cell count (WBC) were significantly higher in the LSS group than in the control group. Blood biomarker data by subgroup in each group are presented in Table 4. In the three subgroups other than the young female subgroup, WBC was significantly higher and CRP tended to be higher in the LSS group than in the control group. In comparison between subgroups, serum creatinine level was significantly lower in both younger and older female groups compared to male groups in both the LSS group and the control group. Furthermore, among female, younger females had significantly lower creatinine levels than older females in both groups.

### 3.4. Correlation Coefficient Between PhenoAgeAccel and Demographic Data

The correlation coefficient between PhenoAgeAccel and demographic data for each subgroup was summarized in Table 5. There was a statistically significant difference in correlation coefficient between PhenoAgeAccel and SMI between the young male (CC: 0.27) and old female groups (CC: −0.25) (*p* = 0.005), and in age between the old male (CC: −0.02) and young female groups (CC: −0.38) (*p* = 0.047) in the LSS group. Although there was no statistically significant difference in BMI, only the old female group showed a trend toward lower calf circumference (CC: −0.29) in the LSS group. Opposite to the findings in the LSS group, BMI demonstrated a weak positive correlation across all groups (CC: 0.08–0.31) in the control group.

**Figure 2:**
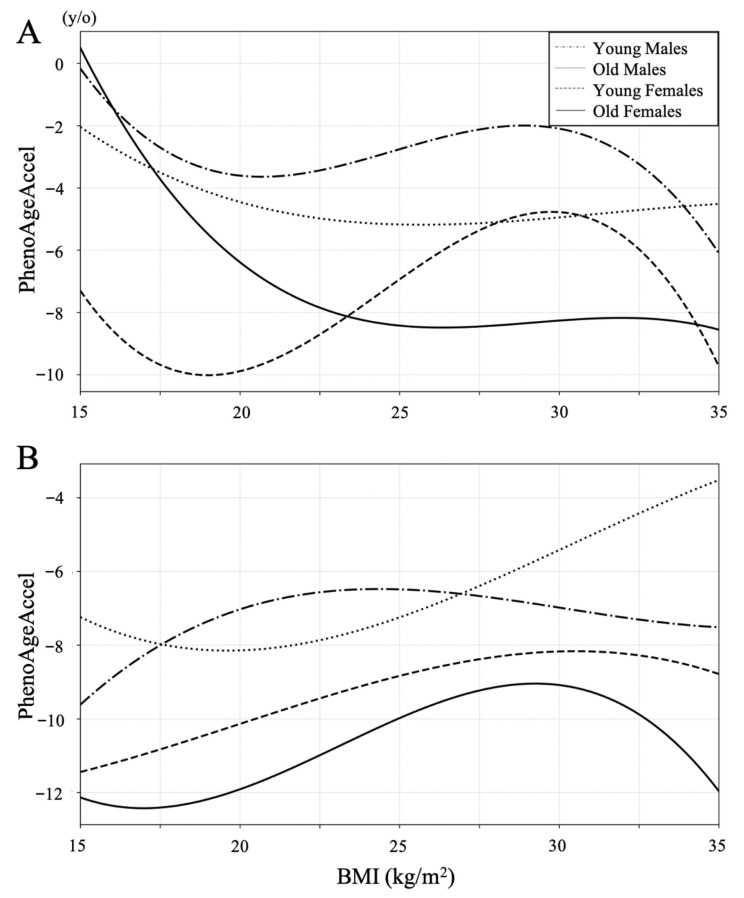
Restricted cubic spline curves between BMI and PhenoAgeAccel in the LSS group (**A**) and the control group (**B**). PhenoAgeAccel decreased with lower BMI in all subgroups of both groups between a BMI of 22 and 30. Lower BMI tended to be associated with lower PhenoAge Accel in the control group, whereas PhenoAge Accel increased with lower BMI below 22 in all subgroups in the LSS group.

Restricted cubic spline curves were constructed to examine the association between PhenoAge acceleration and BMI in both the LSS and the control groups (Figure 2). In the control group, lower BMI tended to be associated with lower PhenoAge acceleration except for the old male group with BMI under 20, whereas PhenoAge acceleration increased with lower BMI below 22 in all subgroups in the LSS group.

## 4. Discussion

In this study, there was a negative correlation between PhenoAgeAccel and SMI only in the old female subgroup in the LSS group. A progressive and generalized skeletal muscle disorder involving the accelerated loss of muscle mass has been defined as sarcopenia, and sarcopenia is associated with increased adverse outcomes including frailty and mortality [25,26]. Incidence of sarcopenia increases with age, and important risk factors of sarcopenia are low activity and bone and joint diseases [26]. These findings are consistent with the high prevalence of sarcopenia and frailty observed in elderly female LSS patients, suggesting that reduced skeletal muscle mass may mediate the association between LSS and accelerated biological aging. Therefore, the lower the SMI, the higher PhenoAge in elderly female patients with LSS accompanied by sarcopenia. While causality cannot be inferred, our results suggest an association between lower BMI and relatively accelerated aging in elderly women with LSS, possibly mediated by sarcopenia and systemic inflammation [8,15].

PhenoAgeAccel increased with lower BMI in the LSS group contrary to the control group. This tendency was particularly pronounced in the old female group, among whom such lower BMI patients were more prevalent. PhenoAgeAccel increased with lower BMI in the LSS group contrary to the control group. This tendency was particularly pronounced in the old female group, among whom such lower BMI patients were more prevalent. Previous reports on the association between BMI and PhenoAgeAccel have indicated that both overweight and underweight were related to high mortality [23]. However, the cutoff for underweight of the study was set at an extreme level of BMI ≤ 18.5, while the entire range of 18.5–25 was regarded as normal. In the present study, among the control group, a BMI around 20 was consistently associated with lower PhenoAge across all subgroups, whereas in patients with LSS, a BMI of 20 already fell within the phase of negative correlation. This suggests that the disadvantage of being underweight is more pronounced in LSS compared with controls. Moreover, previous studies have reported that underweight, particularly in elderly women, has an adverse impact on life expectancy [27]. In aggregate, these findings indicate that both aging and LSS act as factors that exacerbate the negative prognostic impact of being underweight. From this perspective as well, active treatment should be considered beneficial in underweight elderly women with LSS.

In both groups, PhenoAgeAccel was higher in the male subgroups than in the female subgroups. In the LSS group, PhenoAgeAccel was higher in the young male subgroup than in the old male subgroup. In a previous report, males had significantly higher PhenoAge than females using data from a large health examination program, and across different age groups PhenoAgeAccel begins to decline over 70 years old [15]. Regarding previous reports of sex differences in the relationship between biological age and other diseases, females living with HIV over 50 demonstrated decreased epigenetic age acceleration across multiple epigenetic clock measures compared to males, and females also exhibited more accelerated epigenetic aging than males in the patients with Alzheimer’s disease [28,29]. The two best-described explanations for the gender difference in aging are the sex chromosome-linked mechanisms and the hormone-driven differences in biology; however, the gender differences in biological age have been largely ignored and not investigated thoroughly [30]. Therefore, further investigation is needed into the gender differences in PhenoAge and musculoskeletal diseases including LSS.

Inflammatory biomarkers including CRP and WBC were higher in the LSS group than in the control group especially in the three subgroups other than the young female group. Some biomarkers, such as ALP and glucose, exhibited wide variability, possibly reflecting individual differences in metabolic or nutritional status. These findings should be interpreted cautiously. Regarding the relationship between lumbar spine disorders and inflammatory biomarkers, it has been reported that in patients with lumbar disk herniation, serum CRP levels were significantly higher than in control subjects, although still within the normal range, suggesting a potential influence of nerve impingement [31]. In addition, a strong association between CRP levels and pain severity was also reported [32]. However, it is unclear whether this mild inflammatory finding is due to nerve compression, pain, psychological reasons, or other factors that contribute to poor ADL [32,33]. In this study, when compared with the control group, the effects of age and BMI could be excluded. To our knowledge, this is the first study demonstrating elevated inflammatory biomarkers in LSS independent of age and BMI, providing novel evidence linking spinal degenerative disease with systemic biological aging. Further investigations regarding the association between LSS and inflammatory markers are warranted.

This study has several limitations. First, its cross-sectional design precludes causal inference between lumbar spinal stenosis (LSS) and biological aging. Although significant associations were found between PhenoAgeAccel, body composition, and inflammation, the temporal direction of these relationships remains uncertain. Second, despite matching controls for age and BMI, potential confounders such as comorbidities, medications, nutrition, and activity level were unavailable and may have affected the results. Third, this was a single-institution study, and the control cohort consisted of healthy individuals who underwent health check-ups at the same hospital between 2020 and 2023, which may limit generalizability. Finally, although PhenoAge and PhenoAgeAccel are validated markers of frailty and mortality, their clinical relevance in spinal disorders requires further investigation. Future longitudinal, multi-center studies including detailed clinical and lifestyle information are warranted to clarify the causal and mechanistic relationships between LSS and systemic biological aging.

## 5. Conclusions

LSS was associated with relatively accelerated biological aging estimated by PhenoAge, particularly in elderly women with low BMIs. These findings indicate potential links between spinal degeneration, body composition, and systemic aging that require longitudinal validation.

## Figures and Tables

**Table 1:** Comparison of Participant Demographic Data by Study Group.

	LSS Group (n = 208)	Control Group (n = 196)	*p* Value
Gender (Male/Female)	105/103	103/93	0.691
Age (y/o)	70.2 ± 9.3	70.5 ± 8.6	0.835
PhenoAge (y/o)	65.0 ± 10.7	62.0 ± 9.2	0.003
PhenoAgeAccel (y/o)	−5.7 ± 6.5	−8.5 ± 3.7	<0.001
Height (m)	1.60 ± 0.10	1.60 ± 0.09	0.890
Weight (kg)	64.5 ± 13.6	63.4 ± 12.2	0.395
BMI (kg/m^2^)	25.1 ± 4.0	24.6 ± 3.5	0.239

Mean and standard deviation.

**Table 2:** Comparison of Participant Demographic Data by Subgroups.

LSS Group	Young Males (n = 52)	Old Males (n = 53)	Young Females (n = 38)	Old Females (n = 65)	*p* Value
Age (y/o)	62.2 ± 5.6	77.0 ± 4.6	61.2 ± 6.9	77.1 ± 4.5	<0.001
PhenoAge (y/o)	59.9 ± 8.4 *	72.6 ± 6.4 *	52.4 ± 8.3	70.2 ± 7.3 *	<0.001
PhenoAgeAccel (y/o)	−2.9 ± 6.7 *	−4.8 ± 4.4 *	−8.6 ± 7.8	−6.9 ± 5.9 *	<0.001
Height (m)	1.70 ± 0.06	1.64 ± 0.07	1.57 ± 0.05	1.51 ± 0.05	<0.001
Weight (kg)	74.6 ± 12.4	68.0 ± 9.6	61.9 ± 14.4	55.2 ± 9.8	<0.001
BMI (kg/m^2^)	25.9 ± 4.0	25.0 ± 2.7	25.2 ± 5.3	24.3 ± 3.9	0.198
SMI (kg/m^2^)	8.1 ± 1.4	7.4 ± 1.0	6.4 ± 0.8	6.0 ± 0.8	<0.001
**Control Group**	**Young Males** **(n = 51)**	**Old Males** **(n = 52)**	**Young Females** **(n = 36)**	**Old Females** **(n = 57)**	***p* Value**
Age (y/o)	62.9 ± 5.6	77.1 ± 4.7	62.2 ± 6.3	76.6 ± 3.6	<0.001
PhenoAge (y/o)	55.9 ± 6.8	70.3 ± 6.3	52.9 ± 6.7	65.7 ± 4.7	<0.001
PhenoAgeAccel (y/o)	−7.0 ± 2.8	−6.7 ± 4.0	−9.3 ± 3.0	−10.8 ± 3.2	<0.001
Height (m)	1.68 ± 0.06	1.66 ± 0.05	1.57 ± 0.06	1.50 ± 0.04	<0.001
Weight (kg)	72.4 ± 10.9	68.4 ± 9.0	59.9 ± 10.7	53.1 ± 7.5	<0.001
BMI (kg/m^2^)	25.6 ± 3.6	24.9 ± 2.6	24.5 ± 4.3	23.5 ± 3.2	0.016

Mean and standard deviation. *p* value is the result of ANOVA. *: Significant difference compared to the control group.

**Table 3:** Comparison of Participant Blood Biomarker Data by Study Group.

	LSS Group (n = 208)	Control Group (n = 196)	*p* Value
Albumin (g/dL)	4.3 ± 0.3	4.4 ± 0.3	0.008
Creatinine (mg/dL)	0.80 ± 0.23	0.83 ± 0.20	0.315
Glucose (mg/dL)	117 ± 30	112 ± 21	0.036
CRP (mg/dL)	0.37 ± 1.33	0.16 ± 0.17	0.031
Lymp (%)	29.7 ± 8.9	31.5 ± 8.1	0.034
MCV (fl)	91.9 ± 8.0	92.7 ± 4.9	0.205
RDW-CV (%)	13.0 ± 1.1	12.5 ± 0.1	<0.001
ALP (U/L)	74.2 ± 22.5	68.0 ± 17.7	0.002
WBC (10 × 3/μL)	6.20 ± 1.69	5.39 ± 1.36	<0.001

Mean and standard deviation.

**Table 4:** Comparison of Participant Blood Biomarker Data by Subgroups.

LSS Group	Young Males (n = 52)	Old Males (n = 53)	Young Females (n = 38)	Old Females (n = 65)	*p* Value
Albumin (g/dL)	4.3 ± 0.3 *	4.3 ± 0.3	4.5 ± 0.3 *	4.2 ± 0.4 *	<0.001
Creatinine (mg/dL)	0.91 ± 0.23	0.91 ± 0.22	0.64 ± 0.11	0.72 ± 0.21	<0.001
Glucose (mg/dL)	122 ± 42	118 ± 25	108 ± 26	118 ± 24 *	0.164
CRP (mg/dL)	0.26 ± 0.56	0.44 ± 1.81	0.20 ± 0.44	0.49 ± 1.64	0.645
Lymp (%)	29.5 ± 7.9 *	29.5 ± 8.5	29.3 ± 9.7 *	30.3 ± 9.6	0.942
MCV (fl)	90.8 ± 8.8	94.2 ± 4.2	89.2 ± 13.5	92.5 ± 3.5	0.015
RDW-CV (%)	13.0 ± 1.1 *	13.0 ± 0.9 *	12.8 ± 1.7 *	13.0 ± 0.8 *	0.697
ALP (U/L)	71.5 ± 19.2	74.2 ± 25.4	74.1 ± 19.1	76.4 ± 24.3 *	0.716
WBC (10 × 3/μL)	6.32 ± 1.61 *	6.59 ± 1.67 *	5.66 ± 1.17	6.10 ± 1.95 *	0.063
**Control Group**	**Young Males** **(n = 51)**	**Old Males** **(n = 52)**	**Young Females** **(n = 36)**	**Old Females** **(n = 57)**	***p* Value**
Albumin (g/dL)	4.5 ± 0.3	4.4 ± 0.3	4.4 ± 0.2	4.4 ± 0.3	0.003
Creatinine (mg/dL)	0.92 ± 0.15	0.97 ± 0.18	0.69 ± 0.14	0.70 ± 0.13	<0.001
Glucose (mg/dL)	112 ± 20	121 ± 26	106 ± 11	107 ± 17	0.001
CRP (mg/dL)	0.13 ± 0.09	0.17 ± 0.14	0.20 ± 0.23	0.16 ± 0.19	0.219
Lymp (%)	33.1 ± 7.9	29.4 ± 8.2	33.6 ± 6.7	30.7 ± 8.6	0.036
MCV (fl)	92.3 ± 4.1	93.8 ± 3.8	90.7 ± 4.8	93.5 ± 6.0	0.012
RDW-CV (%)	12.5 ± 0.1	12.5 ± 0.1	12.5 ± 0.1	12.5 ± 0.1	0.997
ALP (U/L)	65.7 ± 15.3	68.7 ± 21.2	74.2 ± 17.2	65.5 ± 15.9	0.093
WBC (10 × 3/μL)	5.50 ± 1.38	5.56 ± 1.50	5.29 ± 1.37	5.20 ± 1.18	0.481

Mean and standard deviation. *p* value is the result of ANOVA. *: Significant difference compared to the control group.

**Table 5:** Correlation Coefficient between PhenoAgeAccel and Relevant Variables from Demographic Data by Subgroups.

LSS Group	Young Males (n = 52)	Old Males (n = 53)	Young Females (n = 38)	Old Females (n = 65)	*p* Value
Age	0.10	−0.02 *	−0.38 *	0.03	0.260
PhenoAge	0.73	0.68	0.63	0.78	0.614
Height	0.09	−0.12	−0.06	0.15	0.508
Weight	0.04	−0.11	−0.07	−0.20	0.424
BMI	−0.003	−0.06	−0.09	−0.29	0.189
SMI	0.27 *	0.06	0.09	−0.25 *	0.051
**Control Group**	**Young Males** **(n = 51)**	**Old Males** **(n = 52)**	**Young Females** **(n = 36)**	**Old Females** **(n = 57)**	***p* Value**
Age	0.22	0.03	−0.09	−0.07	0.544
PhenoAge	0.60	0.67	0.36	0.62	0.006
Height	−0.02	0.04	−0.05	−0.12	0.833
Weight	−0.07	0.26	0.27	0.24	0.429
BMI	0.08	0.30	0.29	0.31	0.264

Correlation coefficients were compared across the groups using Fisher’s z-transformation. *p* value is the result of Fisher’s z-transformation. *: Significant difference between the subgroups.

## Data Availability

The data supporting the findings of this study are not publicly available due to institutional privacy restrictions but are available from the corresponding author upon reasonable request.

## Abbreviations

The following abbreviations are used in this manuscript:

LSS	Lumbar spinal stenosis
SMI	Skeletal mass index
BMI	Body mass index
CRP	C-reactive protein
MCV	Mean corpuscular volume
RDW-CV	Red cell distribution width–coefficient of variation
ALP	Alkaline phosphatase
PhenoAgeAccel	Phenotypic age acceleration
ANOVA	Analysis of variance
